# Respiratory Syncytial Virus Prophylaxis: Maternal Vaccination or Long-Acting Monoclonal Antibodies? A Brief Narrative Review of Current Status

**DOI:** 10.3390/vaccines14080673

**Published:** 2026-08-03

**Authors:** Fani Ladomenou, Ioanna-Andriana Psarra, Despoina Gkentzi

**Affiliations:** 1 Department of Paediatrics, Medical School, University of Ioannina, 41510 Ioannina, Greece; faniladomenou@uoi.gr; 2 Department of Paediatrics, Medical School, University of Patras, 26504 Rio, Greece; joannapsarra@upatras.gr

**Keywords:** vaccination, RSV, maternal, monoclonal antibodies

## Abstract

Respiratory syncytial virus (RSV) remains a leading cause of severe lower respiratory tract infection in early infancy, with the highest burden concentrated in the first months of life. Two immunoprophylactic strategies—maternal RSVpreF vaccination and long-acting monoclonal antibodies—now offer effective early life protection, yet differ in biological mechanisms, operational requirements, and programmatic implications. Maternal vaccination induces high-quality neutralizing antibodies that are transferred transplacentally, providing immediate protection from birth and integrating efficiently into antenatal care platforms. However, its effectiveness is constrained by gestational timing, physiological antibody waning, and reduced protection when vaccination occurs shortly before delivery. Long-acting monoclonal antibodies, including nirsevimab and clesrovimab, bypass maternal immune variability and provide standardized, season-long protection across gestational ages, including preterm infants. Economic evaluations demonstrate that both strategies can be cost-effective, though their relative value depends on local epidemiology, pricing, and coverage patterns. Economic outcomes for monoclonal antibodies and maternal vaccination are highly context-dependent and influenced by regional RSV epidemiology, healthcare utilization, product pricing, and implementation capacity, rather than universally favoring one strategy over the other. Global equity considerations remain critical: disparities in antenatal care access, supply chain fragility, and pricing differentials may widen gaps between high- and low-income countries. Remaining evidence gaps—including long-term safety monitoring, durability of protection, viral evolution, and implementation challenges—underscore the need for continued research and careful integration of these tools into national immunization programs.

## 1. Introduction

Respiratory syncytial virus (RSV) is a leading cause of acute lower respiratory tract infection in infants worldwide, with nearly all children experiencing at least one infection by two years of age [1]. Although most infections are mild, RSV can cause severe bronchiolitis and pneumonia, particularly in infants younger than six months. The global burden is substantial: in Europe, RSV accounts for approximately 250,000 hospitalizations annually among children under five years [2], while prospective cohort data indicate that one in 56 otherwise healthy infants is hospitalized with RSV during the first year of life [3]. The highest risk is concentrated in early infancy, with infants younger than three months representing 58.5% of RSV-related ICU admissions [4]. In the United States, 2–3% of infants under three months require hospitalization for RSV each year [5]. Although preterm infants and those with chronic lung disease, congenital heart disease, immunodeficiency or severe cystic fibrosis are at increased risk, a large proportion of hospitalizations occur in previously healthy infants [5].

Together, these epidemiological patterns underscore the urgent need for preventive strategies that provide reliable protection during the earliest months of life. The recent availability of a maternal bivalent prefusion F (RSVpreF) vaccine and long-acting monoclonal antibodies (mAbs) has transformed the prevention landscape, offering two biologically distinct yet potentially complementary approaches. This brief narrative review synthesizes key evidence from randomized trials, real-world evaluations, and economic models to summarize the current landscape of RSV immunoprophylaxis.

This brief narrative review was informed by a structured literature search conducted in PubMed/MEDLINE, Scopus, and WHO IRIS. The search covered the period from January 2018 to June 2026 and used combinations of the following terms: “RSV”, “respiratory syncytial virus”, “maternal vaccination”, “RSVpreF”, “nirsevimab”, “clesrovimab”, “monoclonal antibodies”, “immunoprophylaxis”, “effectiveness”, and “cost-effectiveness”. We included randomized controlled trials, real-world effectiveness studies, national surveillance reports, and economic evaluations relevant to RSV prevention in infants. Articles not reporting primary data, lacking relevance to infant RSV immunoprophylaxis, or published in languages other than English were excluded. Additional references were identified through manual screening of bibliographies of key publications. As this is a narrative review, the aim was to synthesize representative and high-quality evidence rather than to perform a systematic assessment.

## 2. Overview of Studies on Maternal Vaccination Against RSV

### 2.1. Maternal RSV-Associated Vaccination

Maternal immunization with the bivalent prefusion F vaccine represents a major advancement in the prevention of RSV disease during early infancy. In August 2023, the U.S. Food and Drug Administration approved the RSVpreF vaccine (Abrysvo) for administration during pregnancy to prevent RSV-associated lower respiratory tract infection (LRTI) in infants younger than six months [6]. Due to theoretical concerns regarding an increased risk of preterm birth when administered earlier in gestation, U.S. recommendations restrict vaccination to 32–36 6/7 weeks of gestational age [6]. This schedule is endorsed by the Advisory Committee on Immunization Practices, the American College of Obstetricians and Gynecologists, and the Society for Maternal-Fetal Medicine [7,8]. In contrast, the European Medicines Agency authorizes a broader window of 24–36 weeks’ gestation [9]. The vaccine contains a stabilized prefusion RSV F protein antigen, which elicits high titers of RSV-specific neutralizing antibodies in pregnant individuals [10]. These antibodies are actively transferred across the placenta, providing passive protection from birth through the first six months of life [10]. Current recommendations advise against repeat administration of RSVpreF in subsequent pregnancies due to insufficient evidence, and vaccination should be timed to coincide with the RSV season. In the northern hemisphere, administration is recommended from September through January to ensure infant protection during the October–March/April RSV season. Subsequent evidence has, however, proven reassuring regarding the theoretical risk of preterm birth. In the final MATISSE analysis, Madhi et al. reported similar preterm birth rates in vaccine and placebo recipients (5.7% vs. 4.7%), with most events occurring at ≥34 weeks’ gestation and neonatal outcomes comparable across groups [11]. Real-world cohort studies have likewise demonstrated no increased risk of preterm delivery following RSVpreF vaccination, including the large observational analyses by Son et al. and Blauvelt et al., with the latter even suggesting reduced rates of preterm birth among vaccinated individuals [12,13]. Although these findings are reassuring, they should be interpreted in light of important methodological considerations. Randomized trial data provide high internal validity, whereas observational studies may be influenced by residual confounding, differences in healthcare access, and variability in population characteristics. These factors may affect the generalizability of real-world estimates across diverse settings. Continued post-marketing surveillance will be essential to confirm long-term maternal and infant safety across diverse populations and to detect rare outcomes that may not be captured in pre-licensure studies.

### 2.2. MATISSE Phase 3 Randomized Clinical Trial

Robust evidence for the efficacy and safety of maternal RSVpreF vaccination derives from the MATISSE phase 3 randomized, double-blind, placebo-controlled trial conducted across 18 countries [14]. Pregnant individuals aged <49 years received a single 120 µg intramuscular dose of RSVpreF (60 µg each of RSV-A and RSV-B antigens) or placebo between 24 and 36 weeks’ gestation. Among 7358 enrolled participants, 3682 received RSVpreF and delivered 3570 infants, while 3676 received placebo and delivered 3558 infants included in the efficacy analysis [14].

The trial evaluated two primary endpoints: medically attended RSV-associated LRTI (MA-LRTI) and severe MA-LRTI up to 90, 120, 150, and 180 days of life. Efficacy required the lower bound of the confidence interval to exceed 20% (99.5% CI at 90 days; 97.58% CI thereafter). Only the severe MA-LRTI endpoint met this criterion. During the first 90 days, severe MA-LRTI occurred in 6 infants in the vaccine group versus 33 in the placebo group, corresponding to 81.8% efficacy (99.5% CI 40.6–96.3) [12]. By 180 days, efficacy remained high at 69.4% (97.58% CI 44.3–84.1). For MA-LRTI, efficacy at 90 days was 57.1% (99.5% CI 14.7–79.8), but the lower bound did not meet the prespecified threshold. It is important to note that the MATISSE trial was conducted under controlled conditions that may not fully reflect real-world heterogeneity in antenatal care, maternal comorbidities, or timing of vaccination. While the trial provides robust efficacy estimates, its applicability to settings with limited prenatal access or high rates of preterm birth may be more constrained.

Safety outcomes were favorable. Rates of maternal adverse events within one month of vaccination were similar between groups (13.8% vs. 13.1%), and infant adverse events within one month of birth were likewise comparable (37.1% vs. 34.5%) [12]. Reactogenicity was predominantly mild to moderate, and no vaccine-related serious adverse events were identified in mothers, infants, or toddlers up to 24 months.

The distinction between severe MA-LRTI and overall MA-LRTI efficacy has important clinical implications. The vaccine met the predefined success criterion only for severe disease, suggesting that maternal RSVpreF immunization is particularly effective in preventing the most clinically consequential outcomes—those associated with hypoxemia, hospitalization, and intensive care use. In contrast, the lower and statistically non-significant efficacy for all medically attended RSV-LRTI reflects the broader heterogeneity of milder infections, which may be less influenced by transplacentally acquired antibody levels. From a public health perspective, preventing severe disease carries greater weight, as these cases drive the majority of RSV-related morbidity, healthcare utilization, and costs. Nonetheless, the limited efficacy against non-severe medically attended infections indicates that maternal vaccination alone may not fully reduce the overall burden of RSV-associated healthcare encounters. This distinction underscores the complementary role of infant monoclonal antibodies, which provide more uniform protection across the full spectrum of RSV disease severity.

### 2.3. Real-World Effectiveness Evidence

Argentina: Argentina was the first country to introduce maternal RSVpreF vaccination at a national scale, enabling early real-world evaluation. Three independent multicenter studies consistently demonstrated high vaccine effectiveness (VE).

- BERNI study: VE 78.6% (95% CI 62.1–87.9) against RSV-associated hospitalization in infants <3 months and 71.3% (53.3–82.3) up to 6 months; VE against severe RSV-LRTI was 76.9% (45.0–90.3) [15].
- Buenos Aires multicenter cohort: VE 80.8% (62.8–90.5) in infants <3 months and 66.1% (30.1–83.8) in infants <6 months; VE against PICU admission was 87.2% and against prolonged hospitalization 88.6% [16].
- Gentile et al.: adjusted VE 78.7% (51.4–90.7) against RSV-associated hospitalization; infants of vaccinated mothers had significantly shorter oxygen therapy (4 vs. 7 days) and hospital stay (5 vs. 8 days) [17].

United States: A large retrospective case–control study evaluated RSVpreF effectiveness during the 2023–2025 RSV seasons. Maternal vaccination reduced RSV-associated hospitalization by 67.6% (95% CI 33.2–85.4) in infants <90 days and by 74.2% (25.4–93.0) in infants <30 days, with the highest protection observed during the first month of life [18]. Hospitalization due to RSV-associated LRTD was reduced by 69% (95% CI 25.5–88.0).

Scotland: A national population-based case–control study in Scotland further highlighted the importance of timing. Among 27,565 singleton births, maternal vaccination >14 days before delivery was associated with 82.2% effectiveness (95% CI 75.1–87.3) against RSV-associated LRTI hospitalization [19]. Effectiveness remained high in both preterm (<37 weeks; 89.9%) and term infants (≥37 weeks; 81.5%). In contrast, vaccination ≤14 days before delivery yielded non-significant protection (31.7%; 95% CI 15.2–59.5), underscoring the need for adequate time for antibody transfer.

England (UKHSA): A large UK Health Security Agency evaluation including 289,399 infants reported overall VE of 81.3% (95% CI 78.9–83.4%) when vaccination occurred ≥2 weeks before delivery [20]. Protection was highest when vaccination occurred ≥4–5 weeks before birth (~85%) and declined to ~50% when administered within 10–13 days of delivery. Meaningful protection was observed across gestational age groups, including infants born <32 weeks (62%) and 32–37 weeks (70.6%).

Real-world effectiveness studies offer valuable insights but are inherently subject to potential biases, including differences in testing practices, healthcare-seeking behavior, and RSV circulation patterns across regions. These contextual factors may influence effectiveness estimates and should be considered when comparing results across countries.

### 2.4. Parental View/Determination of Vaccine Uptake

Parental acceptance of maternal RSV immunization is generally moderate to high across recent studies, but uptake is shaped by a consistent set of cognitive, emotional, and contextual determinants. The dominant motivator for acceptance is the desire to protect the infant from severe RSV disease and avoid hospitalization, a perception that is strongly reinforced when the vaccine is recommended by a trusted healthcare professional [21]. Trust in clinicians—particularly pediatricians and obstetric providers—plays a central role in shaping positive attitudes, whereas uncertainty or gaps in information may delay or hinder decision-making [22].

Survey data indicate that 76% of pregnant individuals would be likely to accept maternal RSV vaccination if it were recommended by a healthcare provider, and 52% express a preference for maternal vaccination over infant mAb administration strategies [23]. These findings highlight the importance of clinician endorsement and clear communication during prenatal care.

Despite generally favorable attitudes, several barriers continue to limit uptake. The most frequently reported concerns include perceived vaccine novelty, uncertainty about safety, limited awareness of RSV disease burden, and insufficient understanding of available prevention options [24,25].

Overall, the evidence suggests that parental motivation is primarily driven by infant protection, whereas hesitancy stems from informational uncertainty and perceived novelty of RSV immunization strategies. Strengthening communication, improving awareness of RSV disease severity, and ensuring consistent provider recommendations are therefore critical to optimizing maternal RSV vaccine uptake.

## 3. Long-Acting Monoclonal Antibodies for RSV Prevention in Infancy

Long-acting monoclonal antibodies represent the second major pillar of RSV prevention in early life, offering immediate passive immunity through a single intramuscular dose [26,27,28,29,30]. Unlike maternal vaccination, which relies on efficient transplacental antibody transfer, monoclonal antibodies deliver high concentrations of neutralizing antibodies directly to the infant, ensuring protection regardless of gestational age, maternal health status, or timing of birth. Two long-acting mAbs are now available for broad infant use: nirsevimab, licensed in 2022–2023 [28,29], and clesrovimab, approved in 2025 [31,32].

### 3.1. Mechanism of Action and Pharmacologic Design

Both agents target highly conserved epitopes on the prefusion RSV F protein, blocking viral fusion and entry into respiratory epithelial cells [26,27,28,29]. Nirsevimab binds antigenic site Ø, whereas clesrovimab targets antigenic site IV—a region present on both pre- and post-fusion conformations and conserved in >99.8% of circulating RSV strains [29]. Incorporation of Fc-engineering substitutions (YTE triple mutation in clesrovimab; LS mutation in nirsevimab) prolongs binding to the neonatal Fc receptor, extending serum half-life and enabling single-dose, season-long protection.

### 3.2. Nirsevimab: Clinical Efficacy and Real-World Performance

Nirsevimab was the first long-acting mAb to demonstrate high efficacy in healthy preterm and term infants. In the pivotal phase 2b and phase 3 MELODY trials, a single dose reduced medically attended RSV LRTI by 70–75% and RSV-associated hospitalization by 62–78% during the first 150 days of life [28]. A pooled analysis confirmed ~77% efficacy against hospitalization [28]. Safety outcomes were favorable, with adverse events comparable to placebo and no safety signals across >7500 infants.

#### Real-World Effectiveness

Real-world evidence from multiple countries consistently demonstrates substantial reductions in RSV morbidity following universal infant nirsevimab administration. In Galicia (Spain), RSV-related hospitalizations declined by 85% during the first season, with sustained benefits in the second. Similar reductions were observed in Luxembourg and among Alaska Native infants in the United States. A recent meta-analysis of 32 studies confirmed markedly lower odds of RSV hospitalization, ICU admission, and medically attended RSV illness among infants receiving nirsevimab, supporting strong real-world effectiveness across diverse settings [33].

As with maternal vaccination studies, real-world evaluations of nirsevimab may be affected by differences in RSV seasonality, infant healthcare utilization, and local implementation strategies. These sources of heterogeneity should be acknowledged when interpreting cross-country comparisons or pooled effectiveness estimates.

Chile was the first country in the southern hemisphere to implement universal infant nirsevimab immunoprophylaxis at a national scale. The NIRSE-CL retrospective observational study evaluated 154,173 infants during the 2024 RSV season and demonstrated high real-world effectiveness: 76.4% (95% CI 72.6–79.7) against RSV-related LRTI hospitalization, 84.9% (79.5–89.0) against RSV-related ICU admission, and 66.5% (62.0–70.5) against all-cause LRTI hospitalization [34]. The program reduced RSV-related LRTI hospitalizations by 77.5% at the population level, averting 30 cases per 1000 infants, with a number needed to immunize of 35. These findings provide robust evidence of feasibility and substantial public health impact in a middle-income country, highlighting the potential of universal infant immunoprophylaxis in settings with diverse healthcare access and epidemiologic profiles.

### 3.3. Clesrovimab

Clesrovimab (ENFLONSIA™) is a long-acting mAb developed by Merck & Co. and approved in June 2025 in the United States for prevention of RSV LRTI in neonates and infants entering their first RSV season. It binds antigenic site IV of the RSV F protein, a highly conserved epitope across RSV A and B, and incorporates the YTE Fc substitution to extend half-life [35,36].

#### 3.3.1. CLEVER Phase 2b–3 Trial

The pivotal CLEVER trial enrolled 3614 healthy preterm and term infants entering their first RSV season, who were randomly assigned 2:1 to receive a single 105 mg intramuscular dose of clesrovimab or placebo. Over 150 days of follow-up, clesrovimab reduced RSV-associated medically attended LRTI by 60.4% (95% CI 44.1–71.9) and RSV-associated hospitalization by 84.2% (95% CI 66.6–92.6). Serious adverse events occurred at similar frequencies in the clesrovimab and placebo groups, with no overall safety concerns identified [35].

#### 3.3.2. SMART Phase 3 Trial (High-Risk Infants)

The SMART phase 3 trial evaluated clesrovimab in 901 infants and children at increased risk for severe RSV disease, comparing a single 105 mg dose with monthly palivizumab. In this interim analysis, safety outcomes were similar between groups, with no anaphylaxis, hypersensitivity reactions, or safety signals of concern. RSV-associated medically attended LRTI (3.6% vs. 3.0%) and RSV-related hospitalization (1.3% vs. 1.5%) through day 150 were comparable between clesrovimab and palivizumab, indicating non-inferiority in high-risk infants. Serum drug levels and antidrug antibody profiles were consistent with those observed in healthy infants in the CLEVER trial [36].

To date, evidence for clesrovimab derives exclusively from randomized clinical trials, and no real-world effectiveness data are yet available. Post-marketing evaluations during upcoming RSV seasons will therefore be essential to confirm its performance under routine programmatic conditions.

### 3.4. Parental View/Determinants of Monoclonal Antibody Uptake

Parental acceptance of infant administration of long-acting monoclonal antibodies (mAbs) is generally favorable, even though baseline awareness of RSV and its preventive options remains consistently low across settings. Studies report acceptance levels ranging from moderate to very high once parents receive clear, concise information about RSV severity and the protective effect of monoclonal antibodies [16,22,23,24]. A recurring and robust determinant of uptake is trust in healthcare professionals, particularly pediatricians, whose recommendations substantially increase parental confidence and reduce uncertainty [21,22,23,24]. Acceptance is also higher among parents who perceive their infant as vulnerable—such as those with prematurity or prior respiratory illness—and among those who already hold positive attitudes toward routine childhood immunizations [23].

In contrast, hesitancy is driven primarily by safety concerns, unfamiliarity with monoclonal antibodies as a preventive modality, and the perceived novelty of the intervention [22,23,24]. Limited understanding of RSV severity further contributes to uncertainty, especially in contexts where RSV has historically received less public attention than other respiratory infections. Parents consistently express a preference for brief, structured explanations and simple visual materials to support decision-making [23].

Collectively, the evidence indicates that acceptance of long-acting monoclonal antibodies is not hindered by opposition to immunoprophylaxis itself, but rather by knowledge gaps and uncertainty. Targeted education and consistent, clinician-led communication are therefore essential to strengthen parental confidence and optimize uptake of long-acting monoclonal antibody immunization.

## 4. Comparative Effectiveness of Maternal RSVpreF Vaccination Versus Infant Nirsevimab

**Figure 1:**
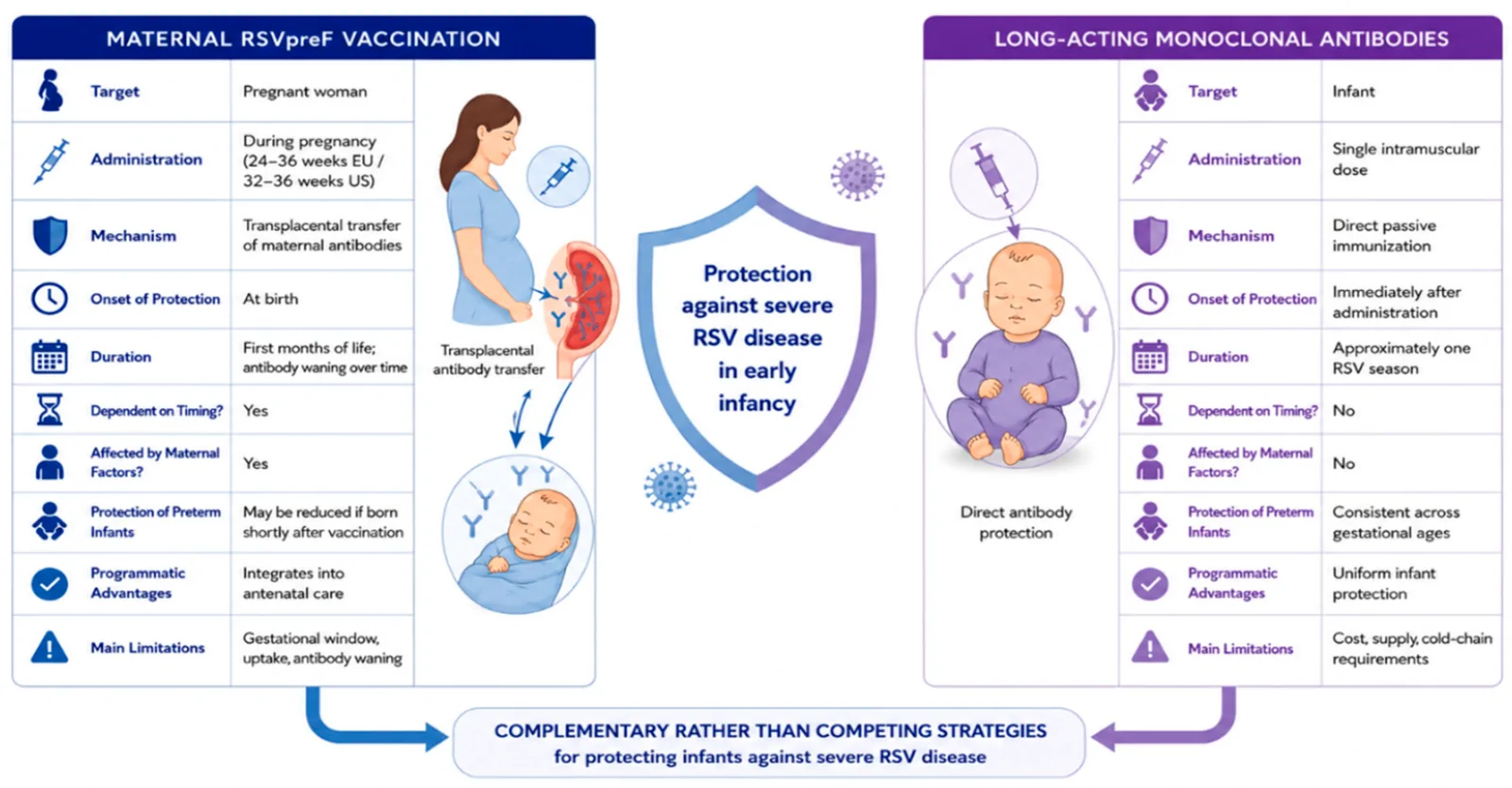
Maternal RSV vaccination and long-acting monoclonal antibodies as complementary strategies for preventing severe RSV disease in early infancy. The figure compares the target population, administration, mechanism of action, onset and duration of protection, key strengths, and limitations of each strategy.

Two large population-based cohort studies from France provide the first real-world head-to-head comparisons between maternal RSVpreF vaccination and infant nirsevimab. Both analyses demonstrated lower risks of RSV-related hospitalization among infants receiving nirsevimab, with consistent reductions in severe outcomes such as PICU admission and ventilatory support [25,26]. Notably, Valtuille et al. showed that when RSVpreF was administered at least eight weeks before delivery, effectiveness approached that of nirsevimab, underscoring the critical importance of optimal timing for transplacental antibody transfer [26]. These findings highlight that while nirsevimab provides robust and consistent protection across all infants, maternal vaccination can achieve comparable effectiveness under conditions of adequate antenatal timing (Figure 1).

It is important to interpret these comparative findings with caution. Head-to-head evidence remains limited, and most available comparisons derive from observational studies that may be influenced by residual confounding, differences in healthcare access, and variability in RSV circulation. As real-world data continue to emerge, conclusions regarding the relative performance of maternal vaccination and monoclonal antibodies should be viewed as evolving. Consistent with this, we emphasize that these two strategies should be considered complementary rather than competing approaches within a broader infant RSV prevention framework.

Beyond these general findings, the choice between these strategies in high-risk infants requires individualized clinical judgment (Table 1). The medical community can be guided by three key pillars: (1) gestational timing and access to prenatal care, where monoclonal antibodies are superior for late-presenting patients or those at high risk of preterm birth by bypassing the need for transplacental transfer; (2) obstetric and fetal risk factors, where direct passive immunization eliminates uncertainty regarding maternal antibody titers; and (3) local epidemiology, where monoclonal antibodies offer a more predictable, season-long ‘umbrella’ of protection in settings with unstable RSV seasonality.

## 5. Discussion

Maternal RSVpreF vaccination and long-acting monoclonal antibodies represent two complementary strategies designed to protect infants during the period of highest vulnerability to severe RSV disease. Maternal immunization offers several biological and programmatic advantages, including broad neutralizing activity against both RSV A and RSV B strains and efficient transplacental IgG transfer that provides immediate protection from birth [27,28,29]. The bivalent formulation induces high-quality antibody responses with evidence of affinity maturation, supporting robust fetal antibody acquisition. Early postnatal mucosal protection through breast milk IgA has also been suggested, although its clinical relevance remains uncertain [37]. Operationally, maternal vaccination integrates seamlessly into established antenatal care pathways and avoids neonatal injections, with safety profiles characterized by mild, transient adverse events [38].

However, the effectiveness of maternal RSVpreF vaccination is inherently constrained by biological and programmatic factors. The recommended gestational window—typically 32–36 6/7 weeks—limits eligibility and may reduce population-level coverage, particularly in settings with late antenatal presentation or high rates of preterm birth [6,7]. Protection is time-limited, as maternally derived antibodies decline physiologically over the first months of life, aligning with the infant’s first RSV season but offering no benefit thereafter. Real-world evidence from the United Kingdom demonstrates substantially reduced effectiveness among infants born within 14 days of maternal vaccination, underscoring the importance of adequate vaccination-to-delivery intervals [19]. Additional uncertainties relate to the absence of universal recommendations for repeat vaccination in subsequent pregnancies and the potential for attenuated immune responses among immunocompromised pregnant individuals. These constraints highlight that maternal vaccination alone cannot guarantee equitable protection for all infants, particularly in settings where antenatal care coverage is inconsistent or where many births occur outside the recommended gestational window.

These limitations highlight the complementary role of long-acting monoclonal antibodies, which bypass maternal immunologic variability and provide direct, standardized protection to all infants regardless of gestational age, maternal health status, or timing of birth. Extended half-life formulations such as nirsevimab and clesrovimab offer uniform efficacy across term and preterm infants, with single-dose regimens that simplify implementation and eliminate pregnancy-related safety concerns [27,28,29,35,36]. Nevertheless, for long-acting monoclonal antibodies such as nirsevimab, a single dose is designed to provide protection confined to the first RSV season, as supported by pharmacokinetic and clinical effectiveness data from phase 2b/3 trials and early real-world evaluations [27,28,29]. At the same time, emerging genomic surveillance data—including early signals of RSV B variants with substitutions at position 208 in the F protein—underscore the need for ongoing molecular monitoring to detect and characterize potential resistance to these agents [39]. Current ACIP recommendations consider maternal vaccination and long-acting mAbs as equivalent options for infants entering their first RSV season, with mAbs preferred when maternal vaccination was not received or occurred <14 days before delivery [40]. Importantly, supply constraints, cold-chain requirements, and procurement costs may disproportionately affect LMICs, where monoclonal antibody access remains limited despite high RSV burden [41]. Ensuring global equity will require coordinated financing mechanisms, predictable supply, and integration into routine newborn services.

Economic evaluations further inform the comparative value of these strategies. Across diverse settings, maternal RSVpreF vaccination demonstrates favorable cost-effectiveness when vaccine prices remain within moderate thresholds and when high antenatal coverage ensures robust transplacental antibody transfer [42,43,44]. Modeling studies from Canada and regional analyses such as Nunavik indicate that maternal vaccination can be economically attractive in contexts with strong antenatal uptake and predictable seasonality [45,46]. In contrast, nirsevimab—and emerging agents such as clesrovimab—tend to yield more favorable cost–utility ratios in settings with high RSV burden, lower product pricing, or universal infant administration at season onset [47]. Several comparative models suggest that monoclonal antibodies may outperform maternal vaccination in cost per QALY gained when maternal uptake is suboptimal or when consistent protection across all infants, including those born preterm or outside the recommended gestational window, is prioritized [47]. Overall, both strategies can be cost-effective, but their relative value is highly context-dependent, shaped by local epidemiology, antenatal care coverage, RSV seasonality, and procurement costs. In LMICs, where antenatal care utilization is variable and cold-chain infrastructure may be limited, hybrid or sequential approaches—maternal vaccination plus infant mAbs for selected groups—may offer the most feasible and equitable path forward.

From a policy and implementation perspective, the choice between maternal RSVpreF vaccination and infant monoclonal antibodies must be tailored to healthcare system characteristics, antenatal care coverage, and local epidemiology. In settings with strong prenatal care infrastructure and predictable RSV seasonality, maternal vaccination may offer efficient integration into existing antenatal platforms. Conversely, in regions with late antenatal presentation, high preterm birth rates, or limited prenatal access, infant monoclonal antibodies provide more reliable and equitable protection. Remaining uncertainties—including long-term safety monitoring, durability of protection, potential viral evolution affecting antibody binding sites, and supply chain stability—underscore the need for adaptive, context-specific decision-making. Clinicians should prioritize timely maternal vaccination when feasible and ensure universal access to monoclonal antibodies for infants who remain unprotected. Policymakers should consider cost-effectiveness within local resource constraints, strengthen procurement and cold-chain systems, and invest in surveillance platforms capable of monitoring RSV epidemiology and product performance. Integrating these tools into national immunization programs will require coordinated planning, sustained financing, and ongoing evaluation to ensure optimal and equitable infant protection.

Taken together, maternal RSVpreF vaccination and infant administration of long-acting mAbs should not be viewed as competing interventions but rather as complementary tools within a broader infant RSV prevention framework. Maternal vaccination provides biologically elegant early protection, whereas monoclonal antibodies ensure uniform coverage across all infants. Importantly, long-term real-world effectiveness data for newer agents such as clesrovimab are not yet available, representing a key evidence gap that will require post-marketing evaluations across multiple RSV seasons. Future research should prioritize more head-to-head comparative studies, including robust comparative effectiveness analyses across diverse healthcare systems where implementation pathways, antenatal care access, and RSV epidemiology differ substantially. A forward-looking agenda must also consider year-round prevention models that align with infant biology rather than calendar-defined seasons—a concept increasingly supported by global epidemiologic instability. Optimal policy will likely require integrated, context-specific approaches that leverage the strengths of each strategy to maximize population-level protection during the critical early months of life. Ultimately, the central message is clear: protecting infants from RSV should not depend on the month they are born, the country in which they live, or the stability of seasonal patterns.

Strengthening genomic surveillance will be essential to detect emerging RSV variants with potential implications for vaccine- or monoclonal antibody-mediated protection. As real-world data accumulate, further comparative studies will be needed to clarify the durability of protection, the performance of hybrid maternal–infant strategies, and the feasibility of implementation across heterogeneous healthcare systems.

An additional priority for future research is the evaluation of RSV preventive strategies in pregnant women living with HIV. Given the high HIV burden in many low- and middle-income countries, understanding how maternal immunosuppression, altered IgG kinetics, and potential differences in placental antibody transfer influence the performance of maternal RSVpreF vaccination is essential. Evidence remains limited, and dedicated studies are needed to ensure that RSV immunoprophylaxis strategies are effective and equitable across diverse maternal health profiles.

### Global Implementation of RSV Immunoprophylaxis

According to the most recent VIEW-Hub data, 45 countries have introduced either maternal RSVpreF vaccination, infant nirsevimab, or both, while an additional ten countries have introduced these products in the private market [48]. Adoption is concentrated in North America, Western Europe, Australia, and parts of Latin America, whereas most countries in Africa and Asia have not yet implemented RSV immunoprophylaxis. Only one country is currently in the planning phase. These patterns highlight substantial global inequities in access to RSV prevention tools and underscore the need for coordinated support mechanisms to facilitate introduction in low- and middle-income countries.

## 6. Conclusions

Maternal RSVpreF vaccination and long-acting monoclonal antibodies represent two complementary strategies that substantially reduce RSV morbidity during early infancy. Maternal vaccination provides biologically elegant protection from birth but is constrained by gestational timing and variability in antibody transfer, whereas monoclonal antibodies offer uniform, season-long coverage across all infants. Real-world evidence confirms high effectiveness for both approaches, though their relative performance varies across healthcare systems, antenatal care access, and RSV epidemiology. From a public health perspective, integrating these tools into national immunization programs will require context-specific decision-making, reliable procurement pathways, and strengthened surveillance to monitor RSV circulation and product performance. Future research should prioritize head-to-head comparative effectiveness studies, evaluation of hybrid maternal–infant strategies, and implementation models suited to regions with unstable seasonality or limited antenatal care access. Ensuring equitable access to both interventions will be essential to maximize infant protection globally.

## Figures and Tables

**Table 1:** Comparative summary of maternal RSVpreF vaccination, nirsevimab, and clesrovimab for infant RSV prevention.

Characteristic	Maternal RSVpreF Vaccination	Nirsevimab (mAb)	Clesrovimab (mAb)
Mechanism of action	Transplacental transfer of maternal neutralizing IgG induced by prefusion F vaccine	Direct administration of high-affinity neutralizing antibody targeting antigenic site Ø	Direct administration of long-acting antibody targeting antigenic site IV (highly conserved)
Timing/Target population	Pregnant individuals (32–36 6/7 weeks US; 24–36 weeks EU)	All infants entering first RSV season	All infants entering first RSV season
Onset of protection	At birth (dependent on timing of vaccination and placental transfer)	Immediately after injection	Immediately after injection
Duration of protection	First 3–6 months of life (antibody waning)	One RSV season (~5 months)	One RSV season (~5 months)
Efficacy (clinical trials)	Severe MA-LRTI: 69–82% (MATISSE)	MA-RSV LRTI: 70–75%; hospitalization 62–78%	MA-RSV LRTI: 60%; hospitalization 84%
Real-world effectiveness	70–85% reduction in RSV hospitalization (Argentina, US, Scotland, UKHSA)	55–85% reduction in RSV hospitalization (Spain, Luxembourg, Alaska); strong meta-analysis support	No real-world data yet available
Safety	Favorable; no increased risk of preterm birth in real-world studies	Favorable; similar to placebo	Favorable; non-inferior to palivizumab in high-risk infants
Advantages	Immediate protection at birth; integrates into antenatal care; avoids neonatal injection	Uniform protection regardless of gestational age; single dose; bypasses maternal variability	Same advantages as nirsevimab; highly conserved epitope; extended half-life
Limitations	Requires adequate timing before delivery; reduced effectiveness if <14 days before birth; limited eligibility window	Protection limited to one season; requires neonatal injection; supply constraints in some regions	No real-world effectiveness yet; implementation pathways still uncertain
Implementation considerations	Depends on antenatal care access and timing; seasonal scheduling required	Universal infant administration at season onset; cold-chain and procurement considerations	Similar to nirsevimab; requires monitoring as programs scale
Current recommendations	Equivalent option to mAbs; preferred if administered ≥14 days before delivery	Equivalent option; preferred if maternal vaccination not received or too close to delivery	Approved for first RSV season; real-world guidance pending

## Data Availability

Data available on request.
